# Comparison of 5-year progression of retinitis pigmentosa involving the posterior pole among siblings by means of SD-OCT: a retrospective study

**DOI:** 10.1186/s12886-018-0817-z

**Published:** 2018-06-26

**Authors:** Leonardo Colombo, Giovanni Montesano, Barbara Sala, Fabio Patelli, Paolo Maltese, Andi Abeshi, Matteo Bertelli, Luca Rossetti

**Affiliations:** 1 Department of Ophthalmology, San Paolo Hospital, University of Milan, Via A. Di Rudinì 8, 20142 Milan, Italy; 2 Optometry and Visual Science, School of Health Sciences, City University London, London, UK; 3 MAGI Human Medical Genetics Institute, Rovereto, Italy

**Keywords:** Disease progression, Ellipsoid zone, Retinitis pigmentosa, SD-OCT, Siblings

## Abstract

**Background:**

The aim of this study is to analyze and compare the progression of photoreceptor atrophy among siblings affected by retinitis pigmentosa by means of spectral SD-OCT.

**Methods:**

Fifty three eyes of 27 patients belonging to 12 family clusters were analyzed. To assess the annual progression rate of photoreceptor atrophy, the ellipsoid zone (EZ) line was measured in OCT sections through the fovea. We used multivariate generalized mixed effects to model the rate of progression and its relation to the initial ellipsoid zone line width.

**Results:**

During our 4.84 years (± 1.44) mean follow up time (range 3–7) 53 eyes were examined. The ellipsoid zone line width declined with a yearly average rate of 76.4 μm (4.16% / year) (*p*-value < 0.0001). Progression rates were poorly correlated within family clusters (*p*-value = 0.23) and showed statistical difference between affected siblings (*p*-value = 0.007). There was no correlation between inter-familiar progression rate and mode of inheritance (*p*-value = 0.98) as well as between age and ellipsoid zone line width among siblings (*p*-value = 0.91).

**Conclusion:**

RP could be extremely heterogeneous even among siblings: an accurate and sensitive method to follow the progression of the disease is fundamental for future development of clinical trials and therapy strategies.

## Background

Retinitis pigmentosa (RP) is a group of inherited retinal disorders leading to vision loss and blindness. It affects approximately 1:4000 individuals with variable modes of inheritance (autosomal dominant, autosomal recessive or X-linked) [1–5].

The peculiarity of RP is its substantial heterogeneity: more than 60 genes are involved, with many possible disease-causing mutations on the same gene, and different clinical outcomes may be linked to the same mutation [5–11].

Despite this heterogeneity, RP patients have some common clinical features: progressive loss of photoreceptors, typically involving the rod system. The characteristic phenotype includes retinal bone-spicule pigmentation, pallor of the optic disk and attenuation of retinal vessel [12].

No universally accepted therapies are currently available for RP but many trials are ongoing evaluating different therapeutic approaches. Neuroprotection, stem cells, gene therapy, optogenetics, electrical stimulation and retinal prosthesis represent possible potential future approaches to slow down the progression of the disease or to restore visual function in patients affected by retinal dystrophies [13–28]. In this light, having deeper knowledge of the mechanisms of disease and more sensitive methods to study its progression is becoming increasingly important.

Most of the studies evaluating RP natural course are based on electroretinography (ERG) and visual field (VF) data. Both methods have an important limitation in evaluating short-term progression due to their high test-retest variability [29–33].

Recently several studies reported the evaluation of Ellipsoid Zone (EZ) line width at SD-OCT as sensitive and reliable marker to detect RP progression [34–38].

The objective of our study was to evaluate disease progression among siblings affected by RP involving the posterior pole and to test whether the progression rate was more similar among subjects within family clusters. For the purpose of our study we measured EZ line changes in a 5-year mean follow up.

## Methods

We retrospectively reviewed data obtained from outpatients of the Retinal Dystrophies department at the University Eye Clinic of San Paolo Hospital in Milan. Among the whole dataset, siblings affected by RP were selected and, upon informed consent, recruited for the study. Within each family siblings were followed for the same period of time and the same number of visits. Diagnosis of RP, clinical and OCT follow up of at least 3 years, stage of disease involving the posterior pole and clearly measurable EZ band on OCT in all past visits were considered as inclusion criteria.

Exclusion criteria included poor OCT scan quality (media opacities, nystagmus), prior vitreo-retinal surgery and not identifiable EZ band on OCT scans.

Diagnosis of RP was based on clinical signs (characteristic bone spicule pigmentation, optic disc pallor, retinal vessel attenuation, visual field constriction, non detectable scotopic electoretinographic waves) and, if available, confirmed by results of genetic analysis. Genetic tests were made in collaboration with MAGI Human Medical Genetics Institute (Rovereto, Italy).

Demographic data and medical history (including the age of diagnosis, the first symptoms onset, inheritance model) were collected from the database. Extent of disease was considered from the date of first diagnosis.

The study was accomplished in compliance with the Declaration of Helsinki and international guidelines.

### Spectral domain optical coherence tomography (SD-OCT)

Retinal imaging was obtained using Spectralis HRA and OCT (Heidelberg Engineering, Heidelberg, Germany). For the study we considered single line scans of 30° and composed of 100 averaged images using the automatic eye tracking software horizontally across the fovea.

All patients meeting the inclusion/exclusion criteria were re-examined. In order to compare OCT scans obtained at the moment of recruitment with previous examinations, since the follow up module was not consistently used, we needed to account for the fact that small displacements of the foveal scan could occur at each visit. Thus, for each follow up visit, we selected the highest quality horizontal OCT scan across the fovea and used each of the selected scans as a reference for a new acquisition at the time of the study, using the progression tool of the Heidelberg Eye Explorer (HEYEX) software.

**Fig. 1:**
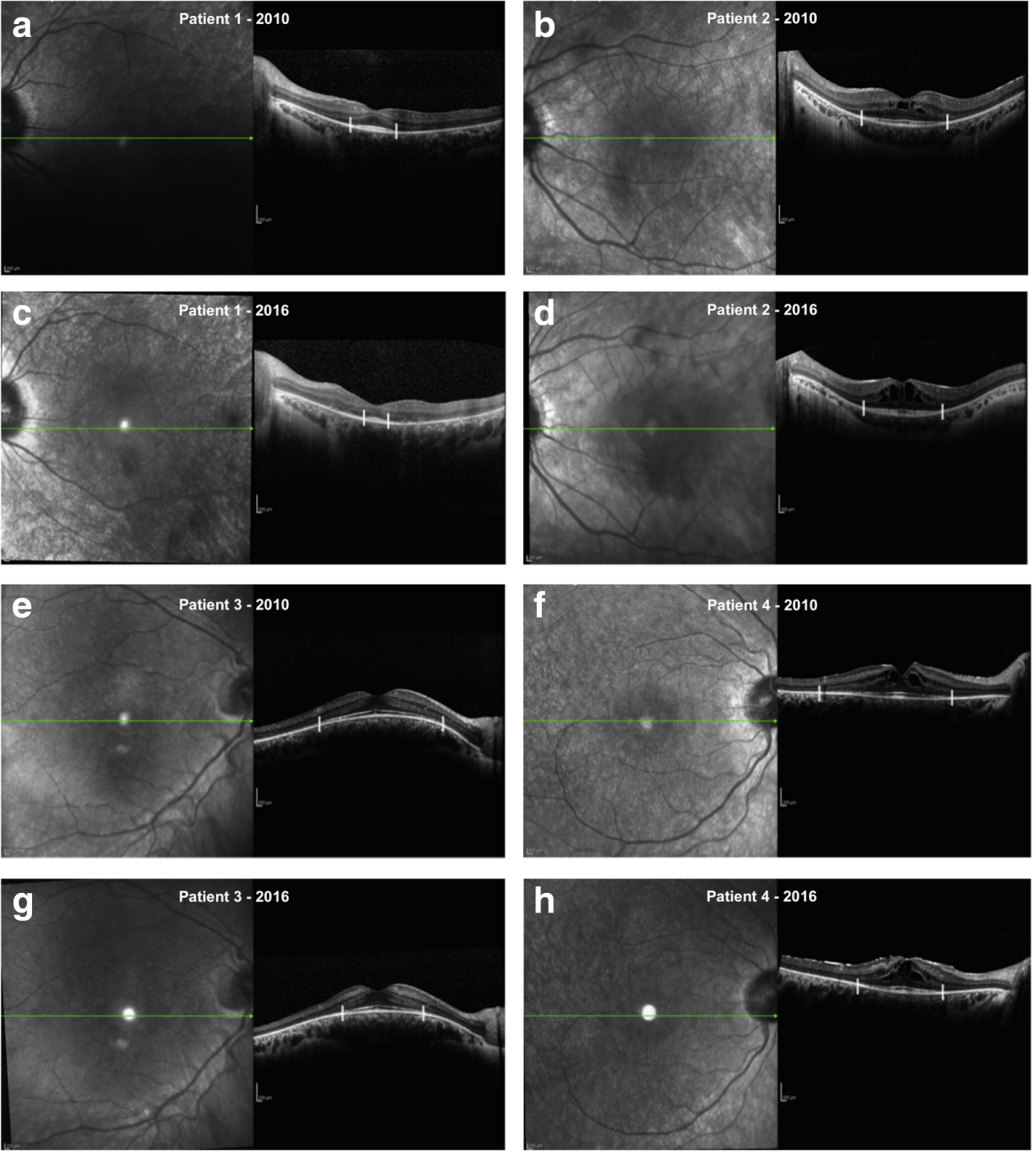
EZ band measurement: EZ limit was considered where the hyperreflective band decline to zero. In **a**, **b** and **e**, **f** horizontal scan passing through the fovea of two couples of siblings affected by RP acquired in 2010, in **c**, **d** and **g**, **h** same scan of same patients acquired 6 years later (2016). Patient in **a** was 17 years old and in 2010 and 23 years old in 2016 (**c**). His brother (**b**) was 18 years old in 2010 and 24 years old in 2016 (**d**). Patient in **e** was 14 years old and in 2010 and 20 years old in 2016 (**g**). His brother (**f**) was 20 years old in 2010 and 26 years old in 2016 (**h**)

EZ line measures were taken manually in a masked fashion using the calliper of the HEYEX software by two experienced OCT-readers as shown in Fig. 1 and the values from the two graders were averaged. EZ limit was considered where the hyperreflective band decline to zero. For the purpose of the study horizontal scans were analyzed: limits were nasal and temporal to the fovea.

Also, the presence/absence of cystoid macular edema (CME) and epiretinal membrane (ERM) was recorded. CME was defined as the presence of hypo-reflective spaces visible on at least two consecutive scans. ERM was defined as the presence of a hyper-reflective line adherent to the inner retina often together with underlying waves in the retinal surface layer due to tractional forces.

All scans were performed with dilated pupils (using 1% tropicamide).

Only OCT scans of good quality (higher than 25 dB) were used for the measurements.

### Statistical analysis

Scans were retrieved in anonymized form from our Spectralis database. As explained in the previous section, in the final dataset, each patient had many progression rate measurements derived from the difference in the EZ line width measured from the study visit and from each of the past sections taken as reference, divided by the elapsed time (in years). Under the assumption that the progression rate (but not the EZ line width) could be considered homogeneous for slight displacements, these measurements allowed estimation of the progression rate across multiple follow up visits.

Calculations were performed using generalized linear mixed models. For the progression rate of the EZ line width, we studied the correlation of the progression rates and the EZ line width at each baseline scan. Nested random effects were used to obtain a multilevel model of the error distribution to account for clustered observations within the same subject and the same family. Specifically, the innermost grouping factor was the single eye: this was used to account for repeated measures performed on the same eye, allowing the calculation of a separate mean rate for each eye considered. The second grouping factor was the single subject: this factor accounted for the correlation between the two eyes. Finally, family was the outermost grouping factor. The last two random effects were used to calculate intraclass correlation coefficients (ICCs) within the same family or subject, in order to assess how family clustering could affect the rate of progression. The ICCs were calculated using the following formula (for the Family random effect):

$$ {ICC}_{Family}=\frac{\sigma_{Family}^2}{\left({\sigma}_{Family}^2+{\sigma}_{Subject}^2+{\sigma}_{Eye}^2+{\varepsilon}^2\right)} $$

where the numerator is the variance attributed to the grouping factor analyzed, while the denominator is the total variance. Group variances are denoted as σ^2^ while the residual errors are denoted as ε^2^. The same formula can be applied to calculate the Subject ICC.

A gamma error distribution with a log link function was used to account for the non-constant variance of the strictly positive value of the progression rate. Such a modelling approach also describes the variation in the rate of change as a proportion of the EZ width in a non-linear fashion.

Similar models were used for the other quantities analyzed, changing the error distribution according to the different variable in study.

## Results

Twenty seven patients were recruited for the study (53 eyes): 9 males (33.3%) and 18 females (66.7%).

Twelve patients (44.5%) were affected by autosomal recessive RP (arRP), 9 (33.3%) by autosomal dominant RP (adRP) and 6 (22.2%) by Usher syndrome type II.

Table 1 summarizes demographic data: mean age was 42.40 years (SD ±13.70) and mean follow up time was 4.84 years (range 3–7 years).

**Table 1:** Demographic data

Demographic	Mean	Standard deviation
Age at baseline (years)	42.40	±13.70
Follow up (years)	4.84	± 1.44
Visual Acuity (decimal)	0.675	± 0.235
Baseline EZ width (microns)	2345.7	±1204.3
Last follow-up visit EZ width (microns)	1945.4	±1123.1

Table 2 reports genetic results of siblings included in the study: seven out of 12 families (58%) harboured a genetic variation probably involved in the phenotype. For two of them (family n°5 and 9), the genetic result was not fully informative considering that only one genetic variant in heterozygous state was found in the autosomal recessive gene *USH2A* that is therefore not sufficient to explain the RP phenotype.

**Table 2:** Genetic results of RP patients included in the study

Family	ID	Sex	Age ranges at baseline	Gene	Transmission	Allele 1 Nucleotide; Amino acid	Allele 2 Nucleotide; Amino acid
1	1	M	50–55	–	AR	–	–
	2	M	45–50	–	AR	–	–
2	3	F	*40–45*	*USH2A*	AR	–	–
	4	M	*35–40*	*USH2A*	AR	–	–
3	5	M	*20–25*	*–*	–	–	–
	6	M	*25–30*	*–*	–	–	–
4	7	F	*65–70*	*–*	AR	–	–
	8	F	*70–74*	*–*	AR	–	–
5	9	F	*20–25*	*USH2A*	AR	c.1841-2A > G	–
	10	F	*30–35*	*USH2A*	AR	c.1841-2A > G	–
6	11	F	*50–55*	*USH2A*	AR	c.1412_1415dup; p.(Asn472Lysfs*2)	c.4124C > T; p.(Ser1375Leu)
	12	M	*50–55*	*USH2A*	AR	c.1412_1415dup; p.(Asn472Lysfs*2)	c.4124C > T; p.(Ser1375Leu)
7	13	F	*45–50*	*NR2E3*	AD	c.166G > A; p.(Gly56Arg)	–
	14	F	*50–55*	*NR2E3*	AD	c.166G > A; p.(Gly56Arg)	–
	15	F	*45–50*	*NR2E3*	AD	c.166G > A; p.(Gly56Arg)	–
	16	F	*45–50*	*NR2E3*	AD	c.166G > A; p.(Gly56Arg)	–
8	17	M	*55–60*	*–*	AR	–	–
	18	F	*50–55*	*–*	AR	–	–
9	19	M	*25–30*	*USH2A*	AR	c.299del; p.(Glu767Serfs*21)	–
	20	F	*35–40*	*USH2A*	AR	c.299del; p.(Glu767Serfs*21)	–
10	21	M	*20–25*	*PRPF8*	AD	c.7007G > C; p.(*2336Serext*41)	–
	22	F	*20–25*	*PRPF8*	AD	c.7007G > C; p.(*2336Serext*41)	–
	23	F	*25–30*	*PRPF8*	AD	c.7007G > C; p.(*2336Serext*41)	–
11	24	F	*35–40*	*USH2A*	AR	c.5776 + 1G > A	c.4758 + 3787_c.6325 + 9314del
	25	F	*45–50*	*USH2A*	AR	c.5776 + 1G > A	c.4758 + 3787_c.6325 + 9314del
12	26	F	*50–55*	*CNGB1*	AR	c.827_834del; p.(Ile276Thrfs*4)	c.2957A > T; p.(Asn986Ile)
	27	F	*45–50*	*CNGB1*	AR	c.827_834del; p.(Ile276Thrfs*4)	c.2957A > T; p.(Asn986Ile)

*ID* patient identification number, *AD* autosomal dominant, *AR* autosomal recessive

**Table 3:** Interclass correlation coefficient

Interclass correlation coeffecient	Value
Family	0.089
Family/Subject	0.175
Family/Subject/Eye	0.068

Compound heterozygous state was confirmed by segregation analysis in AR families n°6, 11 and 12.

Mean baseline EZ width was 2345.7 μm, mean EZ width at last follow up visit was 1945.4 μm.

All patients showed an EZ line width decrease, the estimated mean progression rate was 76.4 μm/year (4.16% per year, *p*-value < 0.0001).

**Fig. 2:**
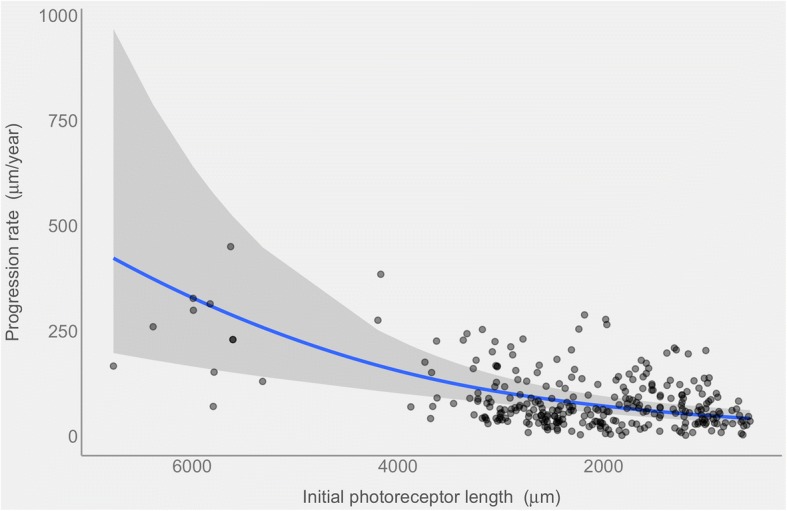
The blue line shows the progression rate (in microns/year) of the photoreceptor length decrease at different baseline lengths (in microns). The curved relationship is a direct consequence of the generalized linear model when displayed on the response scale. The grey band represents the 95% point wise confidence intervals. Single observations are overlaid as semitransparent black dots

Figure 2 shows the relationship between the initial EZ line width in each B-scan and the progression rate over 1 year. The correlation between repeated observations within the same family, the same subject and the same eye was modeled as nested random effects (see Methods). Although only 4 families out of 12 included siblings with different sexes, we tested the effect of sex on the progression rate. Specifically, we used a log-likelihood ratio test to assess whether the inclusion of a random slope in the mixed effect model allowing a change in rate based on subject’s sex for each family cluster provided a significant increase in the goodness of fit. Since the contribution of the sex was not significant (*p* = 0.79) we excluded this factor from the model. The curved relationship derives from the Gamma distribution chosen to model the variance and the log function used to link the mean rate to the predictor. The estimated mean progression rate from the model was 76.4 ± 1.16 μm/year (Mean ± SE) with a 38% ± 0.08% rate reduction for every 10 μm EZ line width.

**Fig. 3:**
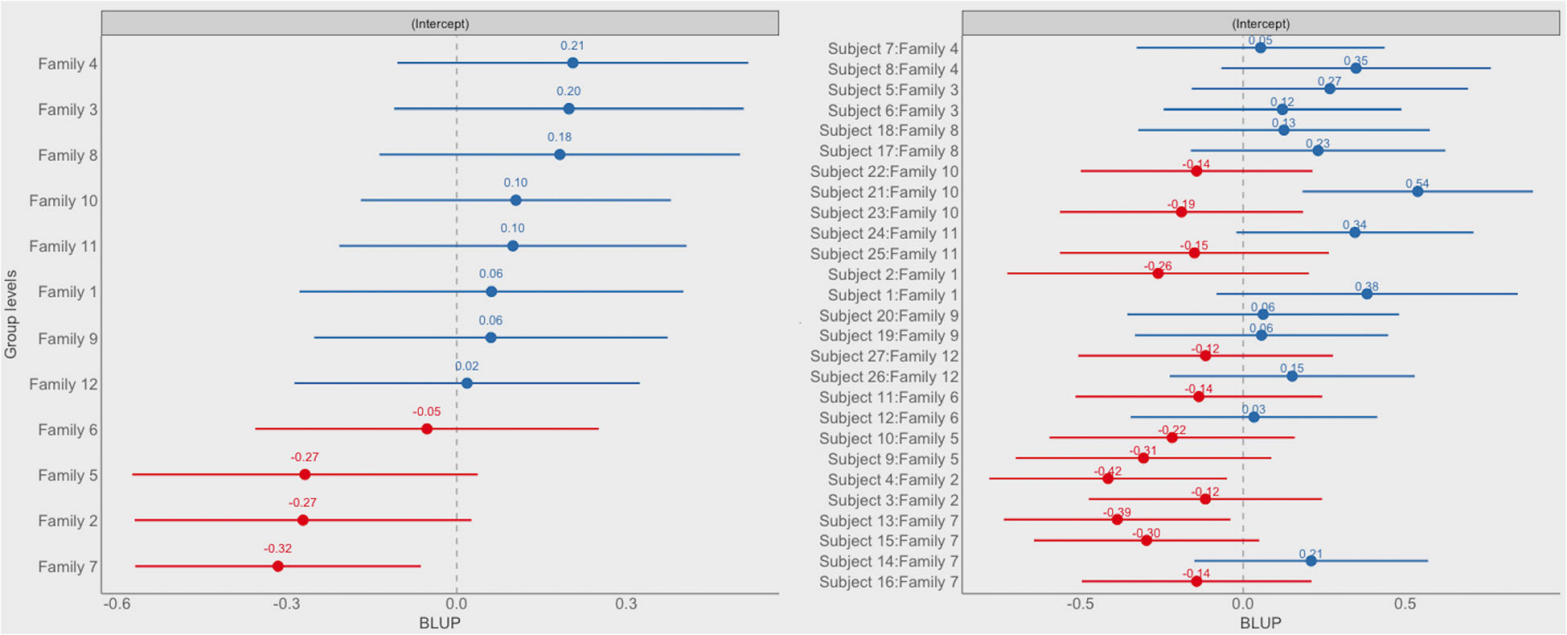
Forest plot of the random effects via BULP (Best Unbiased Linear Predictions) derived from the fitted model of the progression rate. BULP are the best prediction of the group mean (Family or subject), given the observations, from mixed models. The estimated intercept is indicated filled dot and numeric values are reported above each dot. Horizontal lines represent the 95% confidence intervals of the estimates. Reported estimates refer to the intercepts on the log-link function scale of the model and are ordered based on the estimated intercept of the family. Notice how subjects belonging to the same family show very variable estimated intercepts

From the same model we calculated the ICCs of the Family and Subject random effects to assess the contribution of each grouping factor in explaining the variability of the progression rate (Table 3). The highest ICC (0.175) was attributable to the Subject grouping, while the ICC was 0.089 for the Family grouping factor and 0.068 for the Eye grouping factor, suggesting that most of the variance can be ascribed to subject differences, with a lesser contribution from the family cluster and the specific eye, although the values of all ICCs calculated are small in magnitude. Figure 3 depicts the variability at the family and subject level (on the log scale of the link function). Notice the high variability of the single subject around the estimated mean log-rate intercept for each family.

No statistically significant correlation was found between age and EZ line width at baseline (*p*-value = 0.75), even among siblings (*p*-value = 0.91). Similarly, no relationship was found between the progression rate of EZ line width and the mode of inheritance of the disease (adRP versus arRP, *p*-value = 0.98). Of note, Usher syndrome was considered in the group of arRP.

Lastly, the percentage of eyes affected by CME did not change during the whole follow up time (33.3%). The percentage of eyes presenting an epiretinal membrane slightly increased (from 49 to 54.7%).

## Discussion

Our study shows that in our cohort of patients the rate of disease progression among siblings affected by RP, evaluated by means of SD-OCT, is not homogenous.

Clinical and genetic heterogeneity of RP is well known. Natural course of RP could be extremely different among patients, with period of stability that can last years followed by a rapid decrease of visual functions within weeks. The landscape of identified genetic modifications responsible for the disease is becoming increasingly varied, with more than 60 genes involved, each possibly carrying many different mutations [1, 5–12].

The peculiarity of our study is the intra-familiar evaluation of the progression of RP.

Different methods can be used to study the progression of RP. Full field electroretinogram (ffERG) showed its usefulness in quantitating the natural course of disease: limits of ffERG are the lower accuracy in detecting progression in late stages of disease and the high test-retest variability. Because of its features, ffERG is considered to be more reliable when evaluating long-term rather than short-term changes.

High test-retest variability also affects subjective functional evaluations with perimetric tests. On one hand, visual field test has a large dynamic range allowing progression detection even in late stages of disease. On the other hand, both static and kinetic perimetry depend on patient’s collaboration. Moreover, while the former is computer-based, the latter, which is the gold standard in routine follow up of RP patients, might be hampered by operator’s ability and experience in performing the test.

For the purpose of our study we evaluated the progression by means of EZ changes at SD-OCT. Recently, EZ line width has been the subject of several studies evaluating the rate of RP progression [34–38]. The advantages of using EZ band as marker of progression are the low test-retest variability, the accuracy in detecting small changes and the straightforward scan acquisition and analysis. The most important limit is that it cannot be used in early and late stages of the disease. In late RP, when the outer retina is completely atrophic, EZ band is not easily detectable. Conversely, in initial RP cases, when outer retinal atrophy does not involve the posterior pole, the margins of the EZ band cannot be identified within classical OCT scans. However, the boundaries of this latter limitation are being progressively blurred by the introduction of wide field imaging [39].

In general, the mean annual rate of progression is our dataset is comparable with previous studies: the EZ line width reduction was 4.16% per year in accordance with 4.9–10.9% [34, 36, 38] reported in published literature. Similarly to published data, we observed that the progression rate of the EZ band atrophy decreases when the margins of the atrophic retina approach the foveal region [34, 36].

When evaluating the variability of progression rate in our cohort of patients, the highest ICC was attributable to subject grouping: in other terms, being part of the same family cluster, and therefore having the same genetic mutations, does not imply a similar rate of progression (Family ICC = 0.089). Interestingly, we could not find a statistically significant correlation between age and EZ line width among siblings. In other terms, younger siblings in one family cluster could show a more advanced stage of the disease compared to older ones.

However, the lack of any statistically significant interaction between progression rate and mode of inheritance in our study could be explained by the small sample size.

We also considered the percentage of eyes affected by CME or with presence of ERM. In our cohort 33.3% of patients presented CME in accordance with previous reports (13–49%) [40–44].

Literature about ERM incidence in RP patients is controversial with range varying from 1 to 64% [41, 45, 46]. In our cohort the percentage of eyes with evidence of ERM ranged between 49% at baseline and 54.7% at the last follow up exam considered.

## Conclusions

In conclusion, as our data suggested, RP could be extremely heterogeneous even among siblings: an accurate and sensitive method to follow the progression of the disease is fundamental for future development of clinical trials evaluating new therapeutic strategies for RP and others retinal dystrophies. For this purpose, EZ line width evaluation could be considered one of the markers of disease progression. However, the sources of variability identified in our model had overall low ICCs. A more detailed prospective analysis based on follow up measurements on the same location with retinal tracking, modelling the length of the EZ over time instead of the change in rate, might help better characterize the actual dynamics of the progression and reduce the overall variability of the estimates. Furthermore our data suggest that, due to intra-familiar variability, siblings could not offer evident advantages when used as matched controls in treatment trials.

The limitations of our study are represented by the relatively small sample size, the retrospective nature of the study and, as explained above, the fact that we did not use the same section as reference for all follow up examinations.

Prospective studies evaluating EZ band changes with the same reference and combining other variables as control could be helpful in understanding the precise role of family clustering in determining progression rate of RP.

## Abbreviations

adRP: Autosomal Dominant Retinitis Pigmentosa
arRP: autosomal Recessive Retinitis Pigmentosa
CME: Cystoid macular edema
ERG: electroretinography
ERM: Epiretinal Membrane
EZ: Ellipsoid Zone
Hyper-AF: Hyper-Autofluorescence
RP: Retinitis Pigmentosa
SD-OCT: Spectral Domain Optical Coherence Tomography
VF: Visual field
